# Rapid short-pulses of focused ultrasound and microbubbles deliver a range of agent sizes to the brain

**DOI:** 10.1038/s41598-023-33671-5

**Published:** 2023-04-28

**Authors:** William Lim Kee Chang, Tiffany G. Chan, Federica Raguseo, Aishwarya Mishra, Dani Chattenton, Rafael T. M. de Rosales, Nicholas J. Long, Sophie V. Morse

**Affiliations:** 1 Department of Bioengineering, Imperial College London, South Kensington, London, SW7 2BP UK; 2 Department of Chemistry, Imperial College London, Molecular Sciences Research Hub, White City, London, W12 0BZ UK; 3 School of Biomedical Engineering and Imaging Sciences, King’s College London, St Thomas’ Hospital, London, SW1 7EH UK; 4 Division of Radiotherapy and Imaging, The Institute of Cancer Research, Sutton, London, SM2 5NG UK

**Keywords:** Fluorescence imaging, Preclinical research, Drug delivery

## Abstract

Focused ultrasound and microbubbles can non-invasively and locally deliver therapeutics and imaging agents across the blood–brain barrier. Uniform treatment and minimal adverse bioeffects are critical to achieve reliable doses and enable safe routine use of this technique. Towards these aims, we have previously designed a rapid short-pulse ultrasound sequence and used it to deliver a 3 kDa model agent to mouse brains. We observed a homogeneous distribution in delivery and blood–brain barrier closing within 10 min. However, many therapeutics and imaging agents are larger than 3 kDa, such as antibody fragments and antisense oligonucleotides. Here, we evaluate the feasibility of using rapid short-pulses to deliver higher-molecular-weight model agents. 3, 10 and 70 kDa dextrans were successfully delivered to mouse brains, with decreasing doses and more heterogeneous distributions with increasing agent size. Minimal extravasation of endogenous albumin (66.5 kDa) was observed, while immunoglobulin (~ 150 kDa) and PEGylated liposomes (97.9 nm) were not detected. This study indicates that rapid short-pulses are versatile and, at an acoustic pressure of 0.35 MPa, can deliver therapeutics and imaging agents of sizes up to a hydrodynamic diameter between 8 nm (70 kDa dextran) and 11 nm (immunoglobulin). Increasing the acoustic pressure can extend the use of rapid short-pulses to deliver agents beyond this threshold, with little compromise on safety. This study demonstrates the potential for deliveries of higher-molecular-weight therapeutics and imaging agents using rapid short-pulses.

## Introduction

The blood–brain barrier (BBB) poses a considerable challenge for the diagnosis and treatment of brain diseases as therapeutic and imaging agents greater than 400–500 Da and insufficiently lipophilic (≥ 8 hydrogen bonds) are unable to cross the BBB and enter the brain in effective concentrations^1,2^. Net charge and affinities for carrier- or receptor-mediated transport and active efflux transporters are also significant factors in the effectiveness of agent delivery to the brain^3,4^. Currently, more than 98% of small molecule drugs are thought to be BBB-impermeable^5^. Over the past two decades, the combination of focused ultrasound with circulating microbubbles has proven to be a promising method for the transient and localized disruption of the BBB, enabling the delivery of effective concentrations of agents to the brain^6,7^. This technique is commonly used by emitting long pulse sequences of ultrasound, with 10–23.5 ms pulses emitted at a slow rate (≤ 10 Hz) being typical in preclinical and clinical studies^6–20^. However, adverse bioeffects, such as red blood cell extravasation^8,14,21^, transient edema^15,20^ and inflammation^10,13,18^ have been observed in some studies following treatment. Efforts are therefore ongoing to explore the parameter space for improved delivery characteristics and improved biosafety^22–24^. One strategy towards this is the use of short-pulse ultrasound sequences^25–32^.

We have previously designed and tested a rapid short-pulse (RaSP) ultrasound sequence to deliver a 3 kDa fluorescent model agent to the mouse brain^33^. This RaSP sequence comprised of 5 μs (5-cycle) pulses emitted at a 1 MHz center frequency with a pulse repetition of 1.25 kHz, in 10 ms bursts and a burst repetition frequency of 0.5 Hz. At an acoustic pressure of 0.35 MPa, just above the threshold for BBB opening with the RaSP sequence, we found that the model agent was delivered uniformly across the targeted brain tissue within the ultrasound beam. We also found little to no compromise on the dose delivered, when compared with the equivalent long pulse ultrasound sequence. Crucially, RaSP delivered 150 times less acoustic energy to the brain than long pulses and the original BBB permeability was restored within 10 min. Minimal extravasation of endogenous albumin, a protein in the blood implicated in neurotoxic pathways^13,34,35^, was observed and no red blood cell extravasation was detected in the ultrasound-treated brain regions. However, the range of sizes of therapeutic and imaging agents that can be delivered by RaSP has yet to be explored.

In this study, we aimed to extend the favorable delivery characteristics and good biosafety, previously observed when using RaSP, to the delivery of higher-molecular-weight model agents to mouse brains. We also aimed to combine these findings with previous RaSP-mediated deliveries and the subsequent detection of endogenous protein extravasation in mouse brain tissue to indicate size thresholds for delivery by RaSP at a specified pressure. By correlating the delivery characteristics of the model agents with their measured hydrodynamic diameters^36–41^, we demonstrate the potential for RaSP-mediated deliveries of pharmacologically-relevant agents of comparable dimensions.

## Materials and methods

### Animals and study design

All procedures were approved by the UK Home Office and Imperial College London’s Animal Welfare and Ethical Review Body and performed in compliance with the UK Animals (Scientific Procedures) Act 1986 and ARRIVE guidelines^42^.

Animals were held in a room at a controlled temperature (20–24 °C) and humidity (45–65%), and a 12:12 light cycle, including a dawn (30 min) and dusk (30 min) period. Food (RM1 expanded pellets) and water were provided ad libitum.

We targeted the delivery of model agents to the hippocampus due to its importance in learning and memory, and its pathological significance in numerous neurological disorders such as Alzheimer’s disease, schizophrenia and temporal lobe epilepsy^43–45^. Fluorescent dextran was selected as a model agent due to its commercial availability in a range of sizes, its biological and chemical inertness, and its precedent as a tracer in BBB disruption studies, offering good spatial resolution and contrast in brain tissue^8,25,27,32,36,38,40,41,46,47^.

Eleven mice (C57BL/6J, wild-type, 8–17 weeks, female, 20.5 ± 1.0 g; Charles River, Cambridge, UK) were used to assess the delivery profiles of 10 kDa (n = 5) and 70 kDa (n = 6) lysine-fixable Texas Red-labeled dextrans in brains treated with a RaSP sequence and SonoVue^*®*^ microbubbles at 0.35 MPa.

The above deliveries were compared with results from previous deliveries in eleven wild-type mice (C57BL/6J, wild-type, 8–12 weeks, female, 19.6 ± 0.9 g; Charles River, Cambridge, UK) of 3 kDa lysine-fixable Texas Red-labeled dextran (n = 5)^33^ and DiD-PEGylated liposomes (n = 6)^48^. The synthesis and characterization of the DiD-PEGylated liposomes have been previously described^48^. The PEGylated liposomes were chosen as a model large-sized drug delivery agent on account of various liposomal formulations being used clinically to deliver high concentrations of therapeutics (> 10,000 drug molecules per liposome) with low toxicity^49^.

As previously reported, three additional mice (C57BL/6J, wild-type, 8–10 weeks, female, 19.1 ± 0.84 g; Charles River, Cambridge, UK) were used to assess tissue damage following treatment with the rapid short-pulse sequence and delivery of 3 kDa dextran (n = 3)^33^.

To enable comparison between all datasets, an identical ultrasound set-up, pulse sequence and tissue processing method was used, aside from stated exceptions.

A derated ultrasound pressure of 0.35 MPa was selected for deliveries of 10 kDa and 70 kDa dextrans, to match previous deliveries of 3 kDa dextran and liposomes^33,48^. This was determined to be just above the threshold for ultrasound-mediated delivery and demonstrated a good safety profile^33^. In all mice, the left hippocampus was exposed to ultrasound, leaving the right hippocampus as a no-ultrasound control region. The injected dose of 10 kDa dextran was calculated to be the molar equivalent to the previously delivered 3 kDa dextran^33^. A quarter of the molar equivalent dose was used for 70 kDa dextran due to its insolubility in phosphate-buffered saline (PBS).

To assess the extravasation of albumin (66.5 kDa) and immunoglobulin (~ 150 kDa) into the brain, immunostaining was performed on brain slices from experiments in which 3 kDa dextran was delivered. To assess red blood cell extravasation, hematoxylin and eosin (H&E) staining was performed on brain slices from the additional set of mice (n = 3) that were intravenously injected with 3 kDa dextran and SonoVue^*®*^ microbubbles and treated with RaSP.

### Ultrasound set-up and experimental conditions

Mice were anesthetized with 1.5–2.0% vaporized isoflurane (Zoetis UK, Leatherhead, UK) mixed with oxygen (1 L/min) using an anesthetic vaporizer (MSS International, Keighley, UK). Fur was removed from the mouse’s head using an electric trimmer and depilatory cream, before positioning and fixing the mouse’s head within a stereotaxic frame (45° ear bars; World Precision Instruments, Hertfordshire, UK).

**Figure 1:**
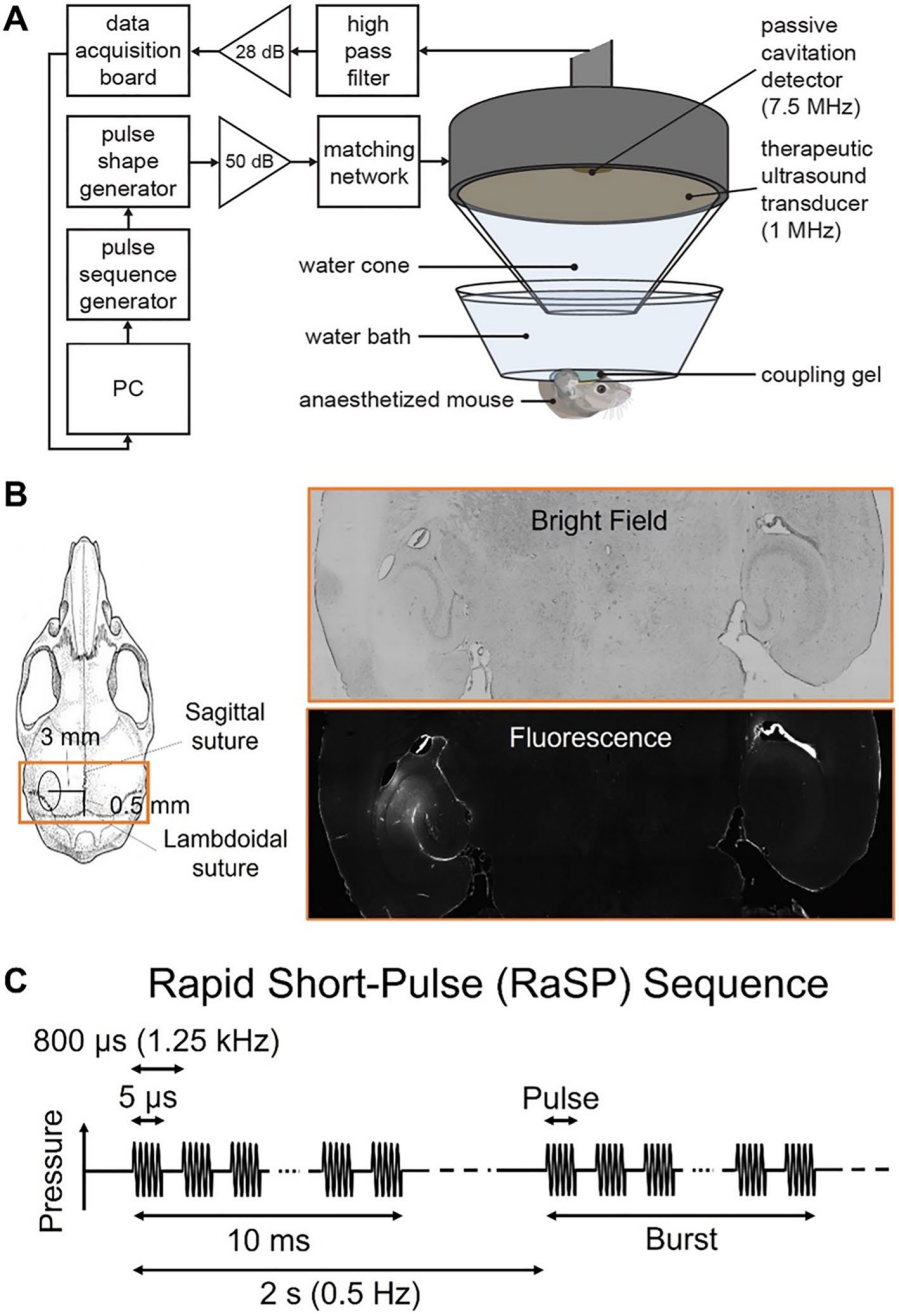
Ultrasound experimental set-up. (**A**) A 1-MHz single-element spherical-segment focused ultrasound transducer was driven by two function generators through an amplifier and a matching network. A passive cavitation detector, placed in the central opening of the transducer such that their foci overlapped, was used to monitor microbubble activity in real time. (**B**) The focal center of the beam was positioned 3 mm inferior to the skull, and 3 mm lateral and 0.5 mm anterior to the intersection of the lambdoidal and sagittal sutures to target the left hippocampus. The right hippocampus, not exposed to ultrasound, was used as a control region to account for background fluorescence within the same mouse. (**C**) A rapid short-pulse (RaSP) ultrasound sequence was emitted to stimulate intravenously administered microbubbles for blood–brain barrier disruption. This sequence consisted of thirteen 5-cycle 1-MHz pulses emitted every 800 μs, grouped into 10 ms bursts emitted every 2 s, with the entire sequence lasting 252 s.

A single-element spherical-segment focused ultrasound transducer (center frequency: 1 MHz; active diameter: 90 mm; focal depth: 60.5 mm; part number: H-198; Sonic Concepts, Bothell, WA, USA) was used for all ultrasound treatments in this study. This was fitted with a cone filled with deionized water and covered with an acoustically transparent parafilm membrane (Fig. 1A). With this configuration, the focal region of the transducer, defined by the full width at half maximum (FWHM), was 1 mm in the elevational dimension, 1.35 mm in the lateral dimension and 20 mm in length. To monitor the real-time microbubble activity within the cerebral vasculature during the ultrasound treatment, a spherically focused passive cavitation detector (PCD) (center frequency: 7.5 MHz; diameter: 12.7 mm; focal length: 76.2 mm; part number: U8423539; Olympus Industrial, Essex, UK) was fitted through the central opening of the transducer and positioned for their foci to coincide. Acoustic emission signals were filtered by a band-pass filter (3–30 MHz; part number: ZABP-16 + ; Mini-Circuits, Brooklyn, NY, USA), amplified by a pre-amplifier (28 dB, Stanford Research Systems, Sunnyvale, CA, USA) and recorded by a 14-bit oscilloscope (GaGe model Octave Express CompuScope; sampling rate: 100 MS/s; DynamicSignals, Lockport, IL, USA).

Ultrasound coupling gel was applied to the mouse’s head and a parafilm membrane-bottomed water bath was placed on top and filled with deionized water. The transducer was lowered to submerge the transducer cone into the water bath.

The left hippocampus was targeted by positioning a 1-mm-thick metal cross in the water bath to align with the lambdoid and sagittal sutures of the mouse’s skull, visible through the intact scalp (Fig. 1B). Using a computer-operated 3D positioning system (Velmex, Bloomfield, NY, USA), the transducer was aligned with the approximate intersection of the metal cross and connected to a pulser-receiver (DPR300; Insidix, Seyssins, France). With the transducer in pulse-echo mode, a 10 mm × 10 mm raster scan was performed to identify the position of the intersection of the metal cross and, therefore, intersection of the sutures. From this established reference point, the centre of the ultrasound beam was positioned to be 3 mm lateral from the sagittal suture, 0.5 mm anterior to the lambdoid suture and 3 mm inferior to the skull, leaving the right hippocampus as a no-ultrasound control.

The rapid short-pulse ultrasound sequence (center frequency: 1 MHz; peak-negative pressure: 0.35 MPa; pulse length: 5-cycles; pulse repetition frequency: 1.25 kHz; burst length: 10 ms; burst repetition frequency: 0.5 MHz; cycles: 126) (Fig. 1C) was generated by two function generators (33500B Series; Agilent Technologies, Santa Clara, CA, USA) to define the pulse shape and pulse sequence. The signal was driven through a 50-dB power amplifier (2100L Electronics and Innovation, Rochester, NY, USA) and an electrical impedance matching network (Sonic Concepts, Bothwell, WA, USA).

As measured and applied in previous work, the peak-negative (rarefactional) acoustic pressure was derated by 11.2 ± 3.2% to the stated 0.35 MPa^33^.

### Delivered compounds and microbubbles

**Figure 2:**
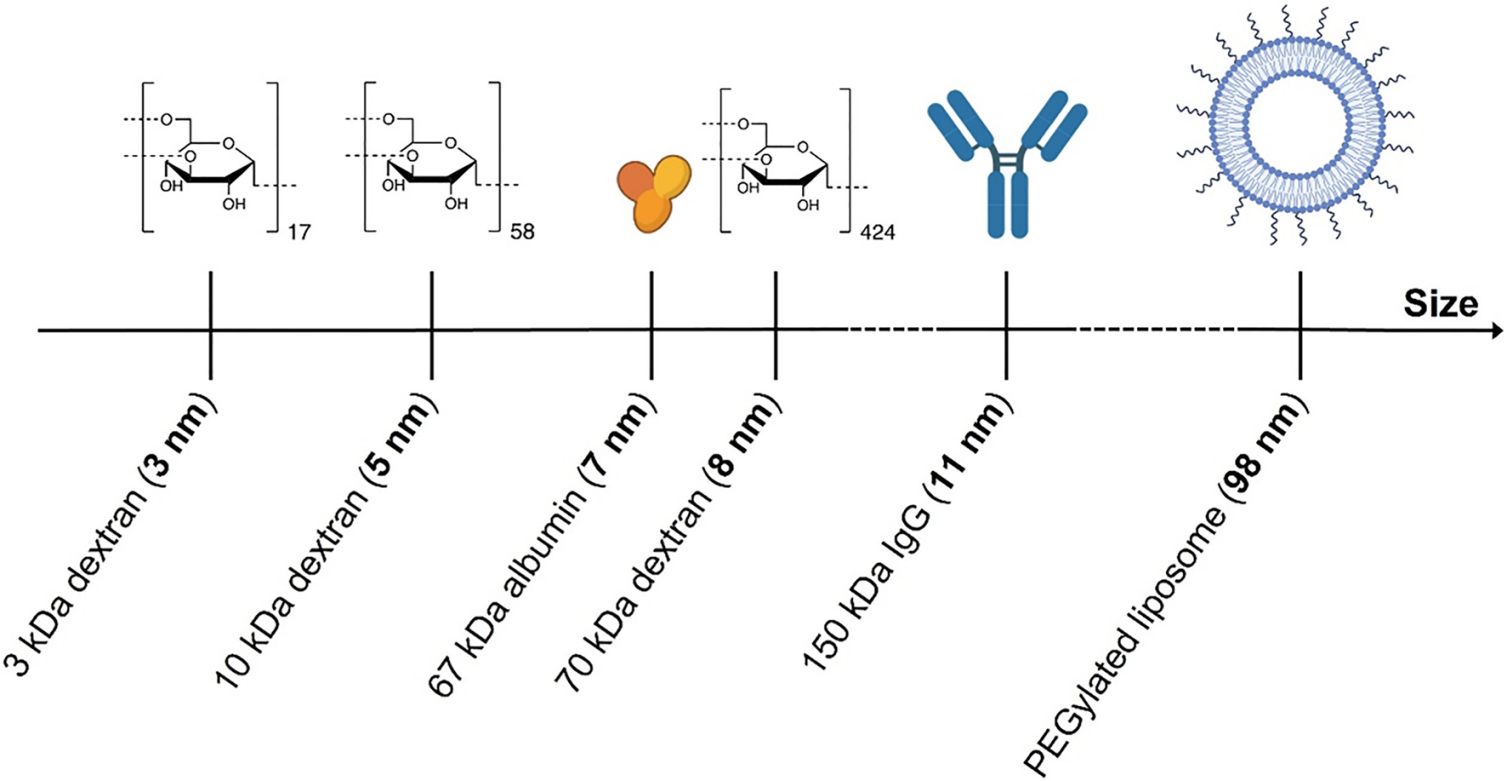
Model agents administered and endogenous proteins assessed for extravasation. Lysine-fixable Texas Red-labeled 3 kDa dextran (hydrodynamic diameter: 3.47 ± 0.65 nm), 10 kDa dextran (hydrodynamic diameter: 5.39 ± 0.9 nm), 70 kDa dextran (hydrodynamic diameter: 8.02 ± 1.60 nm) or DiD-PEGylated liposomes (hydrodynamic diameter: 97.9 ± 2.2 nm)^48^ were administered to mice, immediately prior to their treatment with rapid short-pulses of ultrasound and microbubbles. The extravasation of albumin (hydrodynamic diameter: 7.02 nm)^50^ and immunoglobulin G (hydrodynamic diameter: 10.58 nm)^50^ was subsequently determined by post-treatment histological staining of brain tissue slices. The structures of fluorophores conjugated to administered agents are not shown.

Mice were administered with lysine-fixable Texas Red-conjugated 10 kDa dextran (concentration: 2 mM in PBS; volume: 100 μL; catalog number: D1863, Thermo Fisher Scientific, Invitrogen™, Waltham, MA, USA) or lysine-fixable Texas Red-conjugated 70 kDa dextran (concentration: 0.5 mM in PBS; volume: 100 μL; catalog number: D1864, Thermo Fisher Scientific, Invitrogen™, Waltham, MA, USA) by intravenous tail vein injection with a 30-gauge catheter (Fig. 2).

In previously reported experiments, lysine-fixable Texas Red-conjugated 3 kDa dextran (concentration: 2 mM in PBS; volume: 100 μL; catalog number: D3328, Thermo Fisher Scientific, Invitrogen™, Waltham, MA, USA) and DiD-PEGylated liposomes (lipid concentration: 60.0 ± 0.9 mM, dye concentration: 2.1 ± 0.5 μg/mL (20 dye molecules per liposome); volume: 100 μL)^48^ were injected by intravenous tail vein injection with a 30-gauge catheter.

SonoVue^*®*^ microbubbles (concentration: 8 μL/g body mass; volume: 100 μL) (Bracco, Milan, Italy) were subsequently injected over 30 s, commencing 10 s after the start of the ultrasound sequence in all experiments.

The hydrodynamic diameters of non-fluorescent lysine-fixable biotinylated 3, 10 and 70 kDa dextrans (concentration: 0.1 mg/mL in PBS; catalog numbers: D7135, D1956, D1957, Thermo Fisher Scientific, Invitrogen™, Waltham, MA, USA), were measured at 25 °C using dynamic light scattering (Zetasizer Nano ZS, Malvern Instruments, Malvern, Worcestershire, UK). Hydrodynamic diameters of 3.47 ± 0.65 nm, 5.39 ± 0.9 nm and 8.02 ± 1.60 nm were determined for the 3, 10 and 70 kDa dextrans respectively (Fig. S1). These were in agreement with previously reported measurements^36^. The hydrodynamic diameter of DiD-PEGylated liposomes was reported as 97.9 ± 2.2 nm^48^.

### Histological staining

Following the ultrasound treatment, mice were sacrificed immediately by intraperitoneal administration of an overdose of sodium pentobarbital (concentration: 200 mg/mL; volume: 0.2 mL), followed by transcardial perfusion with heparin in ice-cold PBS (concentration: 20 units/mL; volume: 20 mL; Sigma Aldrich, St. Louis, MO, USA) and fixation of tissues by 10% formalin (neutral buffered; volume: 20 mL; Sigma Aldrich, St. Louis, MO, USA). The brain was excised, further fixed in 10% formalin overnight and then cryoprotected in 30% sucrose in PBS overnight. The brain was subsequently embedded in optimal cutting temperature compound (Agar Scientific, Stansted, Essex, UK) and cryosectioned into 30-μm-thick horizontal slices (CryoStar NX70; Thermo Fisher Scientific, Waltham, MA, USA) with the sample held at a temperature of −12 °C and the blade at −14 °C.

30-μm-thick frozen horizontal brain slices were stained for albumin by application of a rabbit anti-mouse serum albumin primary antibody (1:100 overnight; ab19196; Abcam, Cambridge, UK) and a donkey anti-rabbit IgG H&L Alexa Fluor^®^ 488-conjugated antibody (1:200 for 2 h; ab150073; Abcam, Cambridge, UK). Brain slices (n = 5) were also stained for IgG by application of a donkey anti-mouse IgG H&L Alexa Fluor^®^ 488-conjugated antibody (1:200 for 2 h; ab150105; Abcam, Cambridge, UK).

As previously described, hematoxylin and eosin (H&E) staining was carried out on 6-μm-thick paraffin-embedded horizontal brain slices^33^. These were obtained from an additional set of mice (n = 3) administered with 3 kDa dextran and microbubbles, and treated with the same ultrasound sequence.

### Microscopy and analysis

Brain slice images were acquired by fluorescence microscopy (objective: 10x/0.3 Ph1 EC Plan-Neofluar; working distance = 5.2 mm; Axio Observer; ZEISS; Oberkochen, Germany). Texas Red-labeled dextrans were excited at 562/40 nm and emissions filtered at 624/40 nm, DiD-PEGylated liposomes were excited at 640/30 nm and emissions filtered at 690/50 nm, and Alexa Fluor^®^ 488-labeled staining antibodies were excited at 470/40 nm and emissions filtered at 525/50 nm.

To compare doses of dextrans successfully delivered to brain tissues, normalized optical densities (NODs) were determined for five slices in each brain. For each brain slice, a region of interest (ROI) in the targeted left and control right hippocampi was defined using MATLAB R2019b (Mathworks, Natick, MA, USA). Artifacts—namely blood vessel walls, the choroid plexus and water droplets from the cryosectioning process—were identified and discounted from these ROIs. The fluorescence intensity of pixels with values greater than the mean fluorescence intensity of the control region plus twice its standard deviation were summed in each ROI. The NOD was calculated as the difference between these sums and the mean NOD was determined across the quantified slices for each brain sample. This method was consistent with our previous experiments^14,17,18,33,48^.

To account for variation in the degree of fluorophore labeling between different-sized dextrans (3 kDa: 0.3 mol dye/mol dextran; 10 kDa: 1 mol dye/mol dextran; 70 kDa: 4 mol dye/mol dextran) and the use of a quarter-dose of 70 kDa dextran, weightings were applied to standardize the NODs with respect to 3 kDa dextran (10 kDa dextran: divide NODs by 3; 70 kDa dextran: divide NODs by 3). Delivery was deemed successful if the standardized NOD was at least 1 standard deviation above the mean of the control region. This criterion for successful delivery is consistent with our previous experiments^33^ and has been applied in other studies^25,27^.

To compare the distribution of dextrans in brain tissues, the mean coefficients of variation (COVs) for each brain were determined across the same slices in each brain sample used to calculate the NODs. COVs were calculated as the ratio of the standard deviation to the mean fluorescence pixel intensity in the targeted ROI, providing a combined measure of the area and heterogeneity of fluorescence. This method of quantifying heterogeneity was also consistent with our previous experiments^17,33,48^. The BBB opening area was quantified by measuring areas of fluorescence from the dextran on the microscopy images using ImageJ.

### Statistical analysis

Statistical analysis was performed using GraphPad Prism 5.01 (GraphPad Software, Boston, MA, USA). Shapiro–Wilk test was performed to assess for normal distribution. Upon confirmation of normality of the data, one-way analysis of variance (ANOVA) test was performed for multiple comparisons among NODs and among COVs of different-sized dextrans, followed by post hoc Tukey test. 2-tailed unpaired Student’s *t*-test was performed for the NODs and COVs of different-sized dextrans. *P* < 0.05 was considered statistically significant.

## Results

### Delivering dextrans to the brain

**Figure 3:**
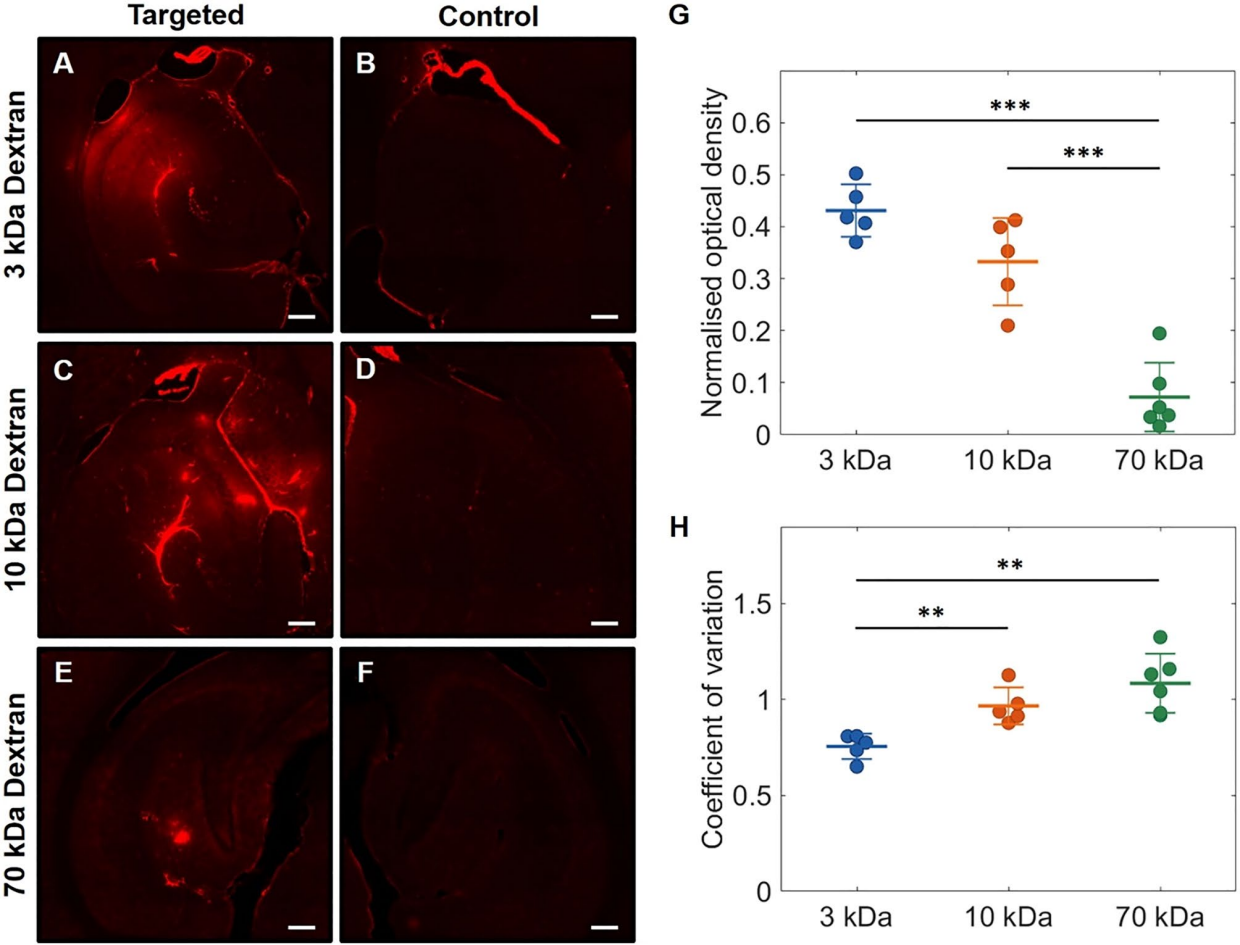
Successful delivery of 3, 10 and 70 kDa dextrans to the mouse brain by RaSP at 0.35 MPa. Fluorescence microscopy images of horizontal mouse brain slices, showing successful RaSP-mediated delivery of (**A**) 3 kDa Texas Red dextran, (**C**) 10 kDa Texas Red dextran, and (**E**) 70 kDa Texas Red dextran (at a quarter of the dose) to the left hippocampus at an acoustic pressure of 0.35 MPa. No fluorescence was observed in the control right hippocampi (**B,D,F**). Fluorescence signals were quantified by calculating the (**G**) normalized optical density (NOD) and (**H**) coefficient of variation (COV) for each mouse administered with 3 kDa dextran (n = 5), 10 kDa dextran (n = 5) and 70 kDa dextran (n = 6). Data are presented as mean ± standard deviation and were analyzed using a two-tailed unpaired Student's *t*-test. **P < 0.01, ***P < 0.001; where *P* < 0.05 was considered statistically significant. Scale bars are 200 µm.

Following treatment of mice with rapid short-pulse ultrasound at 0.35 MPa, fluorescence signals from 10 and 70 kDa Texas Red-labeled dextrans were detected in the left hippocampal region within the focal volume of the ultrasound beam (Fig. 3A,C,E). No fluorescence signals were detected in the corresponding right hippocampus control regions, which were not exposed to ultrasound (Fig. 3B,D,F). These observations were consistent across all mice tested.

However, it was apparent from the microscopy images that the doses delivered to the brain consistently decreased with increasing dextran size and hydrodynamic diameter. This was supported by quantification of the fluorescence signals, with the normalized optical density (NOD), representative of the dose delivered within the region of interest, decreasing with increasing dextran size (Fig. 3G). The mean NOD for 70 kDa dextran (0.07) was 4.6 times less than for 10 kDa dextran (0.33) and 6.0 times less than for 3 kDa dextran (0.43). Statistical significance was observed between the 3 kDa and 70 kDa NODs (*P* < 0.001) and the 10 kDa and 70 kDa NODs (*P* < 0.001), but not between the 3 kDa and 10 kDa NODs (P = 0.0551).

The delivery of larger dextrans also became increasingly restricted to a smaller area. Fluorescence from 3 kDa dextran (Fig. 3A) and 10 kDa Texas Red dextran (Fig. 3C) was observed throughout the volume of the focal beam (defined by the FWHM), while fluorescence from 70 kDa dextran (Fig. 3E) was contained within the area where the local pressure was at least 90% of the maximum focal pressure (Fig. S2). The BBB opening area became increasingly smaller as dextran size increased, with 70 kDa dextran delivered over an area 1.8 and 1.3 times smaller than that of 3 and 10 kDa dextrans respectively (Fig. S3).

The delivery distribution across the tissue also became increasingly heterogeneous. Fluorescence from 10 kDa dextran was noticeably more spot-like across the tissues than with 3 kDa dextran, with regions of concentrated fluorescence spotted unsymmetrically throughout the diffuse area of delivery (Fig. 3C). Fluorescence from 70 kDa dextran was even more punctuated, with only a few discrete areas of fluorescence observed in an unsymmetric, speckled pattern (Fig. 3E). The coefficient of variation (COV), representative of the area of delivery and the heterogeneity of the dose distribution across the region of interest, increased with increasing dextran size (Fig. 3H). The mean COV for 70 kDa dextran (1.08) was 1.1 times greater than for 10 kDa dextran (0.97) and 1.4 times greater than for 3 kDa dextran (0.76), supporting microscopy observations. Statistical significance (*P* < 0.01) was found between 3 and 10 kDa dextran COVs (*P* = 0.0038) and 3 kDa and 70 kDa dextran COVs (*P* = 0.0017), but not between 10 and 70 kDa dextrans (*P* = 0.1735). Trends in the doses delivered and their distributions across brain tissues were in line with the increase in hydrodynamic diameter of the dextrans (Fig. S1).

### Assessing endogenous protein extravasation

**Figure 4:**
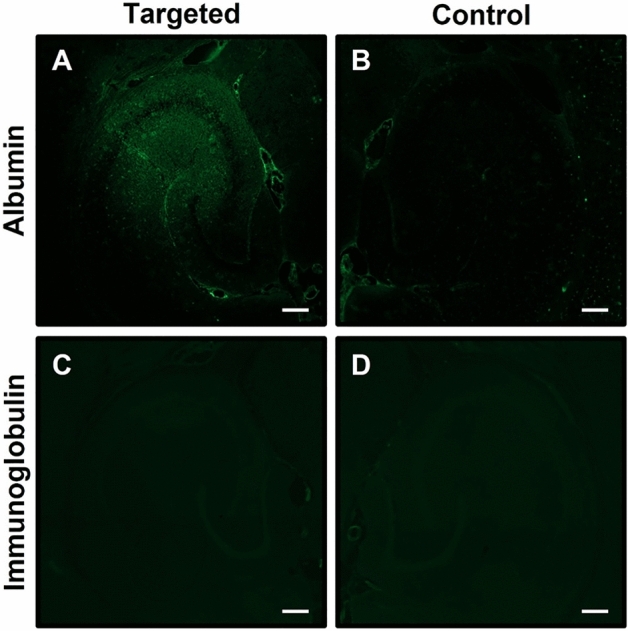
Assessment of endogenous albumin and immunoglobulin extravasation following RaSP treatment at 0.35 MPa. Fluorescence microscopy images of brain slices revealed fluorescence from immunostained endogenous albumin (66.5 kDa) in the (**A**) left hippocampus ROI targeted with RaSP at an acoustic pressure of 0.35 MPa, but not in the (**B**) right hippocampus control ROI of the same brain slice, which was not exposed to ultrasound. No fluorescence from immunostained IgG (150 kDa) was not detected in either the (**C**) targeted left hippocampus or the (**D**) right hippocampus control. Scale bars are 200 µm.

Immunostaining was performed to assess the extravasation of endogenous albumin (66.5 kDa) and immunoglobulin G (IgG; 150 kDa) in the brain following ultrasound treatment with RaSP at 0.35 MPa. Fluorescence signals from immunostained albumin were detected in the targeted left hippocampus (Fig. 4A), but not in the untargeted right hippocampus (Fig. 4B). Conversely, IgG was not detected in either hippocampus (Fig. 4C,D), with no fluorescence signals detected in either region of interest.

### Delivering liposomes to the brain

**Figure 5:**
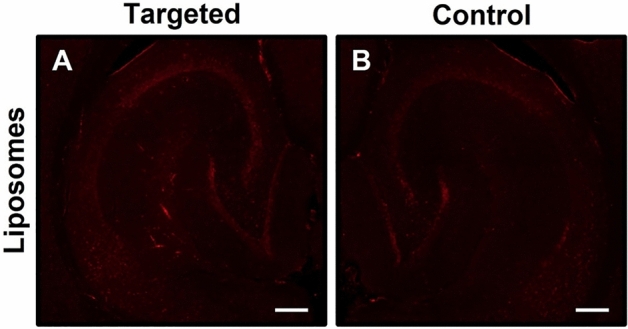
No delivery of DiD-PEGylated liposomes by RaSP at 0.35 MPa. Fluorescence microscopy images of horizontal mouse brain slices showed no DiD fluorescence in both (**A**) the left hippocampus region targeted with ultrasound and (**B**) the right hippocampus control region not exposed to ultrasound. A resultant NOD of 0.068 indicated no delivery of DiD-PEGylated liposomes (97.9 nm diameter) at 0.35 MPa^48^. Scale bars are 200 µm.

DiD fluorescence from DiD-PEGylated liposomes (hydrodynamic diameter: 97.9 nm)^48^ was not detected in either the targeted left hippocampus ROI (Fig. 5A) or the right hippocampus control ROI (Fig. 5B) after sonication of mouse brains using RaSP at an acoustic pressure of 0.35 MPa (n = 6). A resultant NOD of 0.068 indicated no delivery.

### Assessing tissue damage

**Figure 6:**
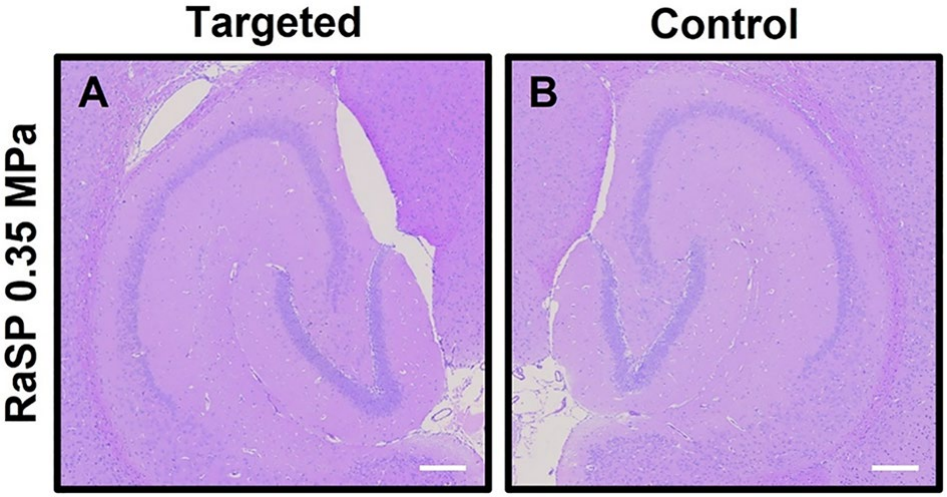
No tissue damage by RaSP at 0.35 MPa. H&E-stained 6-μm-thick paraffin-embedded horizontal brain slices showed no red blood cell extravasation or microvacuolations caused by RaSP emitted at 0.35 MPa^33^. Scale bars are 200 µm.

Following emission of RaSP with an acoustic pressure of 0.35 MPa, no signs of red blood cell extravasation or microvacuolations in the targeted left hippocampus region (Fig. 6A) or the right hippocampus control (Fig. 6B) were found by H&E staining^33^.

## Discussion

### Size threshold of delivery

This work demonstrates the ability that a RaSP ultrasound sequence has in delivering model agents of a range of sizes, with dextrans of increasing size up to and including 70 kDa successfully delivered to mouse brains. Based on our findings, a RaSP sequence emitted at 0.35 MPa can deliver compounds up to a molecular weight between 70 and 150 kDa. Based on the hydrodynamic diameter of 70 kDa dextran, reported here and in the literature^36^, and the hydrodynamic diameter of IgG^50^, this size threshold lies between 8 and approximately 11 nm. This justifies the use of RaSP in future studies for the delivery of therapeutic and imaging agents of comparable sizes, such as antisense oligonucleotides and antibody fragments.

### Size dependency of delivered dose and distribution

In agreement with previous studies using both long-pulse and short-pulse sequences, the larger the model agent, the lower the dose delivered to the mouse brain at a fixed acoustic pressure^14,27,36–41,46,51,52^. The trends we observe in the spatial distribution of agents with increasing size also mirror findings from the aforementioned studies^27,36,38–40,52^. 3 kDa dextran was delivered homogeneously, but 10 and 70 kDa dextrans were delivered in an increasingly heterogeneous pattern with increasing size. The area over which agents were delivered was also smaller with increasing agent size, with 70 kDa delivered only at the center of the beam. This coincides with the pressure differential across the beam, where the pressure output is greatest at the focal point. As would be expected from the known effects of varying pressure on ultrasound-mediated agent deliveries ^21,27,39,40,53–55^, the probability of a suitably large opening for an agent to cross the BBB would therefore increase nearer to the focal point.

The size threshold of delivery for RaSP at 0.35 MPa determined in our study shares similarities with those determined for long-pulse sequences at comparable mechanical indices to the 0.35 used here. Choi et al. reported the delivery of 70 kDa (hydrodynamic diameter: 10.2 nm) using 20 ms long pulses^36^, while Chen et al. observed the delivery of 70 kDa using 1.3 ms long pulses^38^. In both studies, 2000 kDa dextran (hydrodynamic diameter: 54.4 nm) was the smallest agent that was not delivered to the brain. Valdez et al. also showed that 70 kDa dextran could be delivered using 10 ms long pulses, with close to no delivery of 500 kDa dextran (hydrodynamic diameter: 30.6 nm)^40^. Pandit et al. reported the delivery of dextrans up to and including 500 kDa using 10 ms long pulses^41^, while Marty et al. showed the delivery of agents up to and including a hydrodynamic diameter of 65 nm using 3 ms long pulses^37^. Combined, this demonstrates that at a similar acoustic power, long-pulse sequences tendentially disrupt the BBB such that it is permeable to macromolecular agents larger than the threshold for delivery found using RaSP sequences in this study. In our previous work, we have found that liposomes (hydrodynamic diameter: 97.9 nm) do not cross the BBB when using RaSP at 0.35 MPa, but do at a higher pressure of 0.53 MPa^48^. Notably, liposome delivery was possible using the equivalent 10 ms long pulse sequence at 0.35 MPa.

As previously reported, RaSP also leads to a more even deposition of agents across the targeted brain tissues without reducing the total dose delivered to the brain by long pulses^33^. In addition, Zhou et al. recently reported the delivery of an MRI contrast agent in rhesus monkeys treated with the RaSP sequence, using 30 ms pulse lengths^56^. A tenfold increase in the signal enhancement to acoustic energy ratio was achieved, compared with 10 ms long pulses, suggesting a more efficient ultrasound delivery. Long-pulse sequences have proven to be effective at stimulating the vasculature during “on” times and allow the replenishment of microbubbles in the cerebral vasculature during “off” times^25–27,57^. With additional “off” times in-between pulses, the RaSP sequence provides further opportunities to redistribute microbubbles throughout the cerebral vasculature^27,29,30^. By promoting microbubble activity and subsequent extravasation of agents at increased sites within a given volume and time period, disruption of the BBB becomes more uniform across the sonicated region.

### Safety profile

Importantly for clinical translation, the extravasation of endogenous neuroinflammatory IgG (hydrodynamic diameter: 10.58 nm)^50^ following RaSP treatment was not detected in this study but was present when using 10 ms long pulses at the same pressure. H&E staining, following treatment with RaSP at 0.35 MPa, also revealed no extravasation of red blood cells, reported to be approximately 8 µm in diameter and 2 µm thick^58^, while the equivalent long-pulse sequence yielded red blood cell extravasation at multiple sites within the brain tissues^33^. This, as well as the absence of microvacuolations, suggested no tissue damage was caused to the capillary walls by RaSP. However, we also note that parameters used with long-pulse sequences have been successfully optimized to produce favorable safety profiles^9,11,12,59^. Nevertheless, we believe that our direct comparison suggests RaSP may widen the narrow parameter window for delivery without adverse effects, provided comparable doses can be delivered^9,60^.

The lower size threshold determined in this study and the delivery of lower acoustic energies to the brain imply that RaSP induces smaller openings at each site within the microvasculature, compared with the equivalent long pulses. This gentler stimulation reduces the occurrence of high-energy or destructive microbubble activities, thought to cause tissue damage^27,38,53,59,61,62^. Advantageously, smaller openings enable a more stringent selective permeability, with the desired agent able to cross the BBB in effective concentrations, while neurotoxins, pathogens and red blood cells larger than the desired agent continue to be excluded from the brain. The RaSP parameters used in this study have been reported to lead to fast BBB closing within 10 min (for 3 kDa dextran) after ultrasound treatment^33^. This reduces, compared with long-pulse sequences^33,37,63^, the residual time for which the BBB remains disrupted, enabling rapid restoration of normal BBB functioning and, thus, neuroprotection^64^. Enhanced permeability to small molecules is likely to last longer than our reported 10 min extending the time window for crossing into the brain, while normal permeability to larger compounds, such as blood proteins, is likely to be restored in less than 10 minutes^37^, and even faster further away from the focal point^28,63^. Reduced BBB closing times following ultrasound exposure have also been reported in other mouse studies using short pulses of ultrasound^28,32^.

The combination of improving bubble mobility, milder stimulation for a shorter time at each site, and preserving bubble populations in the cerebral microvasculature results in a uniform delivery and good safety profile^29,30^. This is crucial for clinical use, where an unbiased assessment of disease biomarkers in diagnostic applications and even treatment of targeted tissue with reliable doses are essential. Safe ultrasound exposure is also required for sonication at multiple sites across the brain or repeated treatments when monitoring pathology and during treatment programs^12,47,59,65,66^.

### Mechanistic insights

In agreement with findings from long-pulse studies, our observation of a size dependency on agent delivery suggests the predominance of a paracellular pathway in the RaSP-mediated deliveries of 3, 10 and 70 kDa dextrans to the mouse brain^37,38,41,67^. This is converse to the size independency associated with transcytotic pathways^68,69^. Although the delivery of agents larger than 70 kDa by transcytotic pathways has been reported using long pulses of ultrasound and microbubbles^41,70,71^, we did not observe delivery of 98-nm-in-diameter liposomes at 0.35 MPa using RaSP. This suggests large agents may also be reliant on a predominantly paracellular route for their delivery by RaSP, although it may also be that transcellular routes were impeded if the liposomes were larger than transcytotic vesicles^72,73^. However, we note that the effect of pulse length or acoustic pressure on the prevalence of transcellular pathways across the BBB is currently unknown.

We also note that paracellular pathways have been associated with “fast” extravasation into the parenchyma, taking place during the ultrasound treatment, while transcellular pathways have been associated with “slow” extravasation, beginning 5–15 min following treatment^46,74^. Immediate sacrifice of the mice in this study could have favored paracellular routes, while a fast BBB closing time within 10 min following RaSP treatment may also make transcellular routes unfeasible.

### Delivering larger agents

Despite our findings of a size threshold of approximately 8 to 11 nm using RaSP at 0.35 MPa, we stress that short-pulse sequences can also be used to deliver larger agents using higher acoustic pressures^48^. Recently, Batts et al. reported the delivery of a 4.0 MDa adeno-associated virus serotype 9 (hydrodynamic diameter: ~ 25 nm) using a short-pulse sequence at 1.0 MPa^32^. We have also previously demonstrated the delivery of 98 nm liposomes at 0.53 MPa using the same RaSP sequence used in this study^48^. Crucially, despite the increased pressure, a much-reduced number of red blood cells and microvacuolations was detected, compared with the equivalent long pulses at the same pressure. RaSP can therefore be extended to larger agents, such as intact antibodies and nanoparticle formulations, with only a marginal compromise on safety. Consequentially, we believe RaSP offers a larger tolerance than long-pulses for using elevated pressures before impinging on safety and, hence, potentially a larger tolerance for variability in microbubble activity during sonication.

Moreover, we note that once liposomes had crossed the BBB, we observed limited diffusion of the 98 nm liposomes through the parenchyma in the 2 h following treatment^48^. This may be attributed to their inability to pass through pores in the extracellular matrix, measured at 38–64 nm^75^. A more homogenous BBB disruption promoted by RaSP and passage at increased number of sites is therefore crucial to ensure larger agents can sufficiently access pathological sites across the brain tissue.

### Limitations

In this study, we have demonstrated the ability of RaSP to deliver appreciable doses of model agents to mouse brains with good safety profiles. However, we have used a microbubble dose that is 167 times that of the clinical recommended dose for diagnostic imaging applications. We note that a study by McMahon et al. using RaSP and Definity^®^ microbubbles at a 1:125 dilution, with respect to the dosage of SonoVue^®^ used in our study, did not yield any of the expected benefits over long pulses^76^. However, studies by Zhou et al., using a RaSP sequence with 30 µs pulses and a similar SonoVue^®^ microbubble dose to our study, found enhanced BBB permeability over the equivalent long-pulse sequence and no hemorrhage or edema^56^. In future studies, we aim to systematically investigate the effect of microbubble dose, type and dispersity on the delivery of agents using RaSP. Consideration must also be given to how the size threshold observed in this study and safety profile will differ between organisms and for brain diseases in which BBB integrity is compromised, such as Alzheimer’s disease, Parkinson’s disease and glioblastoma.

To enable their measurement by dynamic light scattering, the hydrodynamic diameters reported for the dextran model agents are those of non-fluorescent biotinylated analogs. Differences in their three-dimensional folded structures, as well as differing degrees of fluorophore labeling may therefore lead to discrepancies between the reported sizes of agents and actual sizes of agents delivered in vivo. We have also standardized the NOD values based on differences in the degree to which the dextrans are labeled with the fluorophore and the dose administered. However, quenching effects have not been accounted for in this study. We stress that the implications of this study are not absolute, with agent size not always dictating delivery profiles^77^. As with long-pulse treatments, the parameters used for the delivery of any agent using RaSP must be optimized in consideration of its physical and pharmacological properties and its intended application.

## Conclusions

In this study, we have demonstrated that a rapid short-pulse (RaSP) ultrasound sequence emitted at 0.35 MPa can deliver model agents up to and including a molecular weight of 70 kDa to mouse brains. This size threshold corresponds to a hydrodynamic diameter between 8 and approximately 11 nm. Mirroring studies with long pulses of ultrasound, increasingly larger agents were delivered to the brain in smaller doses, with a smaller area of delivery and an increasingly heterogeneous distribution. As RaSP was designed to gently stimulate the cerebral microvasculature and increase microbubble mobility for a more uniform distribution of microbubble activity, the observed size limit may arise from smaller individual openings in the blood–brain barrier. Larger macromolecular agents, such as 98 nm liposomes, can be delivered to mouse brains by RaSP at an increased pressure of 0.53 MPa. At both pressures, RaSP yielded a good safety profile, demonstrating the potential of this sequence to deliver therapeutics and imaging agents of a range of sizes to the brain.

## Supplementary Information

Supplementary Information.

## Data Availability

Original data are available from the authors upon request.
