# Supplementary material for: Rapid short-pulses of focused ultrasound and microbubbles deliver a range of agent sizes to the brain
Source: Sci Rep. 2023 Apr 28;13:6963. doi: 10.1038/s41598-023-33671-5 (PMC10147927; doi:10.1038/s41598-023-33671-5)
Supplement: Supplementary file 1 — Supplementary Information. [file 41598_2023_33671_MOESM1_ESM.docx]

**Rapid Short-Pulses of Focused Ultrasound and Microbubbles Deliver a Range of Agent Sizes to the Brain**

William Lim Kee Chang^1,2^, Tiffany G. Chan^1,2^, Federica Raguseo^2^, Aishwarya Mishra^3^, Dani Chattenton^1,4^, Rafael T. M. de Rosales^3^, Nicholas J. Long^2^ and Sophie V. Morse^1,*^

**Supplementary Information**

**Method for Dynamic Light Scattering**

Non-fluorescent lysine-fixable biotinylated 3 kDa, 10 kDa and 70 kDa dextrans (catalog numbers: D7135, D1956, D1957, Thermo Fisher Scientific, Invitrogen™, Waltham, MA, USA) were dissolved in phosphate-buffered saline (concentration: 10 mM, Sigma-Aldrich, Burlington, MA, USA) and diluted to a concentration of 0.1 mg/mL. The solutions were filtered and transferred into polystyrene clear cuvettes (catalog number: 14-955-125; Fisher Scientific, Fisherbrand™, Pittsburgh, PA, USA).

Dynamic light scattering (Zetasizer Nano ZS, Malvern Instruments, Malvern, Worcestershire, UK) measurements were performed on the solutions at 25 °C. Results were analyzed via the built-in software (CUMIN method). Mean hydrodynamic diameters and standard deviations were extracted by fitting the data from each size of dextran to a Gaussian distribution in Python, according to the following equation:

$$y=y_{max}e^{\left( -0.5\left( \frac{x-mean}{standard deviation} \right)^{2} \right)}$$

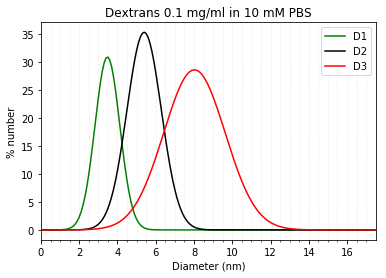


**Figure S1.** Hydrodynamic diameters determined by dynamic light scattering. Mean hydrodynamic diameters and standard deviations of lysine-fixable biotinylated 3, 10 and 70 kDa dextrans were obtained by fitting dynamic light scattering data to a Gaussian distribution. D1 = 3 kDa dextran, D2 = 10 kDa dextran and D3 = 70 kDa dextran.

**Area of Delivery with Increasing Dextran Size**


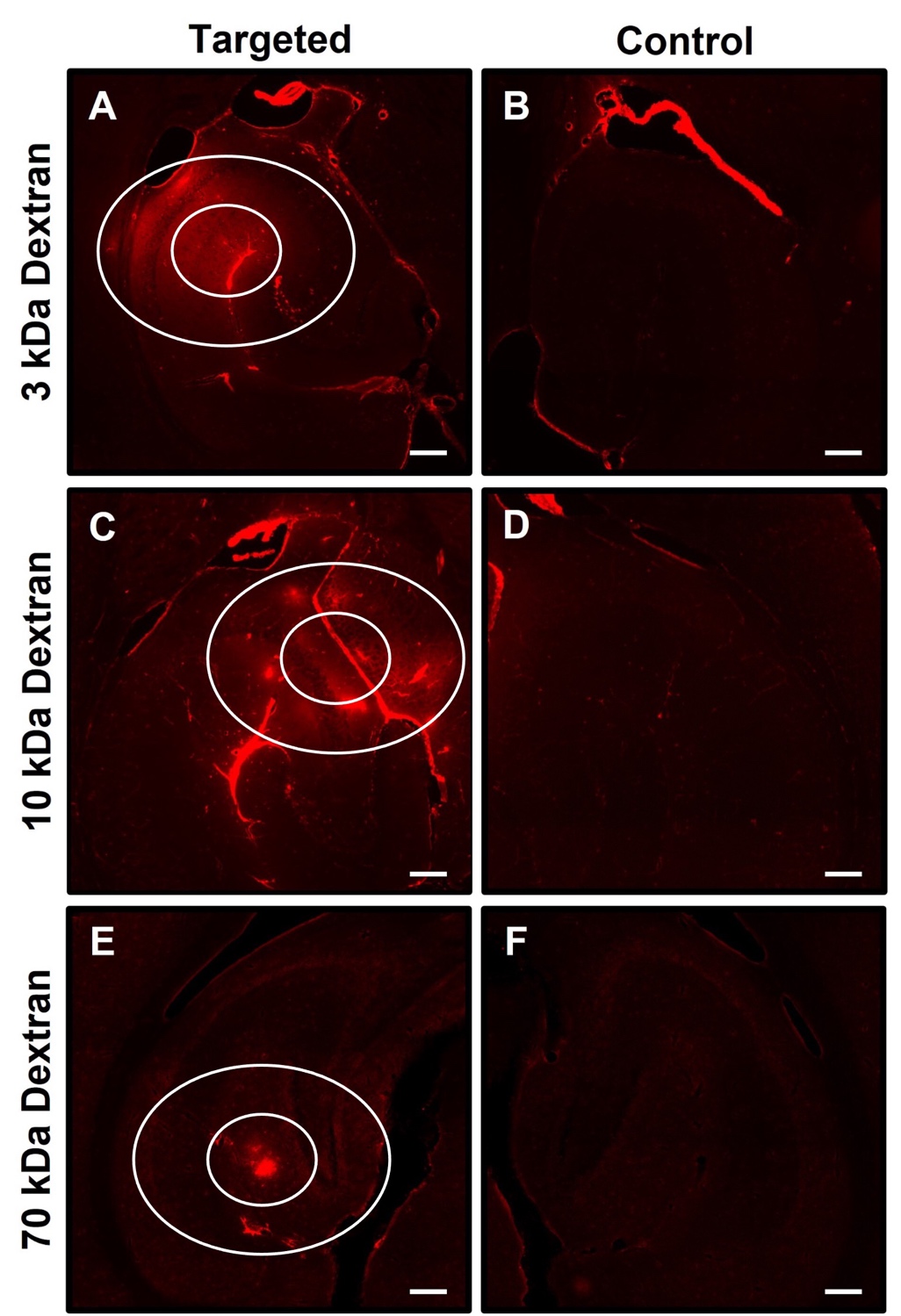


**Figure S2.** Delivery of agents close to the size threshold limit are restricted to the focal point of the ultrasound beam with rapid short-pulses at 0.35 MPa. Microscopy images of horizontal mouse brain slices (Fig. 3) overlaid with the outline (white) of the 1 MHz focused ultrasound beam emitting as defined by the full width at half maximum (FWHM; outer boundary) and the full width at 90 % maximum (FW90; inner boundary) of the output peak-negative pressure of 0.35 MPa (FWHM: 1 mm in the elevational dimension, 1.35 mm in the lateral dimension; FW90: 0.48 mm in the elevational dimension, 0.57 mm in the lateral dimension). The center of the beam is positioned at approximately center of the area of delivery. Following treatment with RaSP at 0.35 MPa, fluorescence from (**A**) 3 kDa Texas Red dextran and (**C**) 10 kDa Texas Red dextran was present throughout the entire focal volume while fluorescence from (**E**) 70 kDa Texas Red dextran (at a quarter of the dose) was restricted to the region defined by the FW90. No fluorescence was observed in the control right hippocampi (**B**, **D**, **F**), which were not exposed to the ultrasound beam. Scale bars are 200 µm.


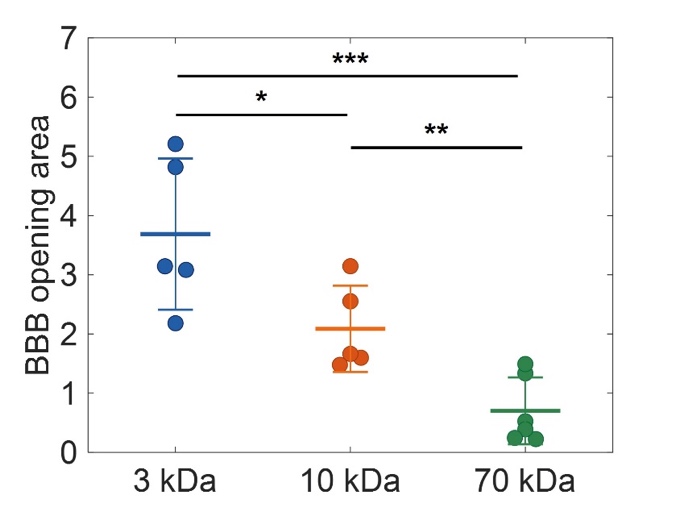


**Figure S3.** Blood-brain barrier opening area decreases with increasing agent size. The area of opening was determined by measuring the areas of fluorescence from the dextrans using ImageJ. The larger the dextran, the smaller the proportion of the ultrasound beam within which it could be delivered to the brain, with 70 kDa dextran delivered over an area 1.8 and 1.3 times smaller than that of 3 kDa and 10 kDa dextrans respectively. Data is presented as mean ± standard deviation and were analyzed by 2-tailed unpaired Student’s *t*-test. * = P < 0.05, ** = P < 0.01, *** = P < 0.001; where P < 0.05 was considered statistically significant.
